# Dietary behaviours and related lifestyles according to the presence or absence of skipping breakfast in Japanese adults: the JPHC-NEXT study

**DOI:** 10.1017/S1368980023000010

**Published:** 2023-06

**Authors:** Chika Okada, Hiroyasu Iso, Kazumasa Yamagishi, Ai Ikeda, Mitsumasa Umesawa, Isao Muraki, Nobufumi Yasuda, Tadahiro Kato, Isao Saito, Kazuhiko Arima, Takayuki Nishimura, Kozo Tanno, Kiyomi Sakata, Atsushi Goto, Taiki Yamaji, Motoki Iwasaki, Taichi Shimazu, Manami Inoue, Norie Sawada, Shoichiro Tsugane

**Affiliations:** 1 Public Health, Department of Social Medicine, Osaka University Graduate School of Medicine, 2-2 Yamadaoka, Suita, Osaka 565-0871, Japan; 2 Department of Public Health, Medicine, Faculty of Medicine, and Health Services Research and Development Center, University of Tsukuba, Ibaraki, Japan; 3 Ibaraki Western Medical Center, Chikusei, Japan; 4 Department of Public Health, Juntendo University Graduate School of Medicine, Bunkyo, Tokyo, Japan; 5 Department of Public Health, Dokkyo Medical University, School of Medicine, Tochigi, Japan; 6 Department of Public Health, Kochi University Medical School, Kochi, Japan; 7 Center for Education and Educational Research, Faculty of Education, Ehime University, Matsuyama, Japan; 8 Department of Public Health and Epidemiology, Faculty of Medicine, Oita University, Oita, Japan; 9 Department of Public Health, Nagasaki University Graduate School of Biomedical Sciences, Nagasaki, Japan; 10 Department of Hygiene and Preventive Medicine, Iwate Medical University, Iwate, Japan; 11 Epidemiology and Prevention Group, Center for Public Health Sciences, National Cancer Center, Tokyo, Japan

**Keywords:** Breakfast, Dietary behaviour, Lifestyle, Cross-sectional study

## Abstract

**Objective:**

To assess dietary behaviours and related lifestyles according to the presence or absence of skipping breakfast.

**Design:**

We analysed the cross-sectional data from a baseline survey of a large-scale population-based cohort study in Japan conducted in 2011–2016. Participants provided information on dietary behaviours and lifestyles through a self-administered questionnaire. Skipping breakfast was defined as not eating breakfast at least once a week and was classified according to the frequency of skipping breakfast as 1–2, 3–4 or ≥5 times/week.

**Setting:**

Sixteen municipalities in seven prefectural areas across Japan under the Japan Public Health Centre-based prospective study for the Next Generation.

**Participants:**

112 785 residents (51 952 males and 60 833 females) aged 40–74 years.

**Results:**

After adjustment for age, socio-demographic status, drinking status and smoking status, individuals who skipped breakfast at least once a week, compared with those who ate breakfast every day, were more likely to have adverse dietary behaviours such as frequent eating out (multivariable OR = 2·08, 95 % CI (1·96, 2·21) in males and 2·15, 95 % CI (1·99, 2·33) in females), frequent eating instant foods (1·89, 95 % CI (1·77, 2·01) in males and 1·72, 95 % CI (1·56, 1·89) in females). They had late bedtime (1·85, 95 % CI (1·75, 1·95) in males and 1·98, 95 % CI (1·86, 2·11) in females) and living alone (2·37, 95 % CI (2·17, 2·58) in males and 2·02, 95 % CI (1·83, 2·21) in females), using the logistic regression model.

**Conclusions:**

Both adult males and females who skipped breakfast were likely to eat out, to have a dietary habit of eating instant foods and have lifestyles such as late bedtime and living alone than those who ate breakfast.

Eating breakfast may be an important dietary behaviour for cardiometabolic health. Several previous cross-sectional studies showed that individuals who skipped breakfast seemed to have an increased prevalence of obesity^(1–5)^, abdominal obesity^(3)^, dyslipidaemia^(3)^, higher serum insulin^(3)^ and high blood pressure^(3)^, and worse health-related quality of life^(2)^. A randomised crossover trial of ten normal-weight females who underwent 2 weeks of eating or skipping breakfast separated by 2-week intervals showed that skipping breakfast resulted in higher fasting total and LDL cholesterol levels^(6)^. Furthermore, prospective studies also showed the association of skipping breakfast with weight gain^(7)^, abdominal obesity^(8,9)^, obesity^(8)^, metabolic syndrome^(8)^, type 2 diabetes^(10,11)^, dyslipidaemia^(9)^, higher fasting insulin^(9)^, risk of CVD^(12,13)^ and total mortality^(14)^. However, the dietary behaviour of skipping breakfast has increased overtime: 14 % to 25 % from 1965 to 1991 for American adults^(15)^, 15·2 % to 27·4 % from 1986–1990 to 2004–2007 for Germany children and adolescents^(16)^ and increased from 2002 to 2010 for adolescents among eleven countries (Belgium, France, German, Croatia, Spain, Poland, Russian Federation, Ukraine, Latvia, Lithuania and Norway), respectively^(17)^.

We consider that the key components of dietary behaviours are when, where and what individuals eat and that these components may be influenced by factors of daily life rhythms and living arrangements, as well as the food culture and system. Therefore, to effect improvements in the public nutritional issue of skipping breakfast, it may be necessary to identify relevant factors such as other dietary behaviours and lifestyles factors related to breakfast intake and take a comprehensive approach, rather than simply targeting skipping breakfast. We assumed that bedtime could affect waking time, and which could affect the habit of breakfast intake, and that living alone could also affect it. To our knowledge, however, no evidence exists to support this hypothesis.

The aim of the present study was to assess selected dietary behaviours (overeating, eating quickly, eating out, and eating instant foods) and related lifestyles (bedtime and living arrangement) according to the presence or absence of skipping breakfast, using cross-sectional data from a recent large-scale population-based cohort study in Japan.

## Methods

### Study population

The Japan Public Health Centre-based prospective study for the Next Generation (JPHC-NEXT study) was initiated in 2011, and the baseline survey was carried out until 2016, and included sixteen municipalities in seven prefectural areas across Japan, namely the Ninohe/Karumai area (Ninohe City and Karumai Town in Iwate Prefecture), Yokote area (Yokote City in Akita Prefecture), Saku area (Saku City, Sakuho Town, Koumi Town, Minamimaki Village, Minamiaiki Village, Kitaaiki Village and Kawakami Village in Nagano Prefecture), Chikusei area (Chikusei City in Ibaraki Prefecture), Konan/Aki area (Kagami and Noichi districts in Konan City and Aki City in Kochi Prefecture), Ozu area (Ozu City in Ehime Prefecture) and Unzen/Minamishimabara area (Unzen City and Minamishimabara City in Nagasaki Prefecture). The survey areas were chosen based on geographical distribution, size and feasibility. The detailed protocol of the JPHC-NEXT Study has been published elsewhere^(18)^. In brief, a self-administered questionnaire was distributed to all residents of the sixteen target municipalities who were asked to report about their socio-demographic attributes, personal medical history, lifestyles, smoking status and drinking status and diet. A total of 114 054 residents (52 554 males and 61 500 females) aged 40–74 years returned the questionnaire. We excluded 1269 participants with incomplete information on breakfast intake, leaving 51 952 males (98·9 %) and 60 833 females (98·9 %) for the present cross-sectional analysis.

### Skipping breakfast

Participants responded about the average frequency of eating breakfast during the past year in a self-administered questionnaire for the following question: ‘How often do you have breakfast?’. The six response categories were less than once a month, 1 to 3 times/month, 1 to 2 times/week, 3 to 4 times/week, 5 to 6 times/week, or every day. In the present study, participants who had breakfast less than once a month, 1 to 3 times/month and 1 to 2 times/week were combined into one category because of the limited numbers: 1864 (3·0 %), 1335 (2·1 %) and 1712 (2·7 %) in males, and 950 (1·6 %), 814 (1·3 %) and 1521 (2·5 %) in females, respectively. Skipping breakfast was defined as not eating breakfast at least once a week and the frequency of skipping breakfast was classified as 1–2 times/week (sometimes), 3–4 times/week (often) and ≥5 times/week (usually). Those who ate breakfast every day were regarded as the reference group.

### Dietary behaviours and lifestyle factors

Participants filled out the average status of dietary behaviours during the past year as follows: (1) overeating (yes or no); (2) speed of eating (very fast, slightly fast, normal, slightly slow or very slow); (3) the frequency of eating out, including lunch box ‘bento’ and rice balls purchased at the store, (less than once a month, 1 to 3 times/month, 1 to 2 times/week, 3 to 4 times/week, 5 to 6 times/week or every day) and (4) the frequency of eating instant foods, e.g. Chinese noodle, cup noodle and retort foods (same six categories as eating out). Participants also provided information concerning bedtime (before 7 p.m., 8 p.m., 9 p.m., 10 p.m., 11 p.m., midnight, 1 a.m., 2 a.m., 3 a.m., 4 a.m. or irregular) and whether they lived alone (yes or no). We assessed these factors combined into binary variables as follows: speed of eating (very fast/slightly fast *v*. normal/slightly slow/very slow), the frequency of eating out and eating instant foods (<3 *v*. ≥3 times/week) and bedtime (before 11 p.m. *v*. 11 p.m. or later and irregular).

### Statistical analyses

Of the 112 785 participants, we excluded individuals with missing values for dietary behaviours (*n* 897 for overeating; 210 for eating quickly; 1505 for eating out and 1301 for eating instant foods) and lifestyle factors (*n* 508 for late bedtime and none for living alone) for each analysis.

We used the chi-square test to compare the sex-specific proportions of covariates and behaviour variables. The OR and 95 % CI of dietary behaviours (overeating, eating quickly, eating out and eating instant foods) and lifestyles (late bedtime and living alone) according to the frequency of skipping breakfast were estimated by logistic regression models. The estimates were adjusted for the following factors: age (continuous), area (nine population cohorts), education levels (junior high school, high school, junior college/vocational school or college graduates and higher), drinking status (never, former or current), smoking status (never, former or current), annual household income (0–2990, 3000–5990, 6000–8990 or ≥9000 thousand Japanese yen) and health examinations in the last year (yes or no). When these covariates had missing values, we used dummy variables. All statistical analyses were performed using SAS version 9.4 software (SAS Institute Inc.). All statistical tests were two-tailed and *P* values <0·05 were regarded as significant.

## Results

The characteristics of participants according to the frequency of skipping breakfast are presented in Table 1. In middle-aged and older adults in the present study, 17·2 % of males and 12·6 % of females skipped breakfast. Participants who skipped breakfast were younger and more current smokers in both sexes, and tended to be more current drinkers among females, compared with those who ate breakfast every day.

**Table 1:** Sex-specific characteristics of participants according to the frequency of breakfast intake

The frequency of skipping breakfast)	Males										*P* for difference	Females										*P* for difference
	Eating breakfast		Skipping breakfast		Sometimes		Often		Usually			Eating breakfast		Skipping breakfast		Sometimes		Often		Usually		
	(0/week)				(1–2 /week)		(3–4 /week)		(≥5 /week)			(0/week)				(1–2 /week)		(3–4 /week)		(≥5 /week)		
	*n*	%	*n*	%	*n*	%	*n*	%	*n*	%		*n*	%	*n*	%	*n*	%	*n*	%	*n*	%	
No. of participants	42 999	82·8	8953	17·2	2402	4·6	1640	3·2	4911	9·5		53 179	87·4	7654	12·6	2576	4·2	1793	2·9	3285	5·4	
	Mean	sd	Mean	sd	Mean	sd	Mean	sd	Mean	sd		Mean	sd	Mean	sd	Mean	sd	Mean	sd	Mean	sd	
Age	59·7	9·3	53·1	8·9	53·6	8·9	53·9	9·1	52·5	8·7	<0·001	59·1	9·5	53·5	8·8	53·9	8·8	53·9	8·9	53·0	8·8	<0·001
	%		%		%		%		%			%		%		%		%		%		
40–49	17·3		39·8		36·8		36·8		42·3		<0·001	19·6		37·1		34·9		35·6		39·6		<0·001
50–59	26·2		33·8		34·0		32·1		34·3			26·8		35·5		36·9		34·5		34·9		
60–69	40·3		22·4		25·2		26·2		19·8			37·8		23·2		23·7		25·9		21·4		
70–74	16·1		4·0		4·0		4·9		3·6			15·8		4·2		4·5		4·0		4·1		
Smoking status																						
Never	20·6		12·7		16·7		13·9		10·3		<0·001	85·7		62·6		72·9		66·0		52·8		<0·001
Former	49·1		30·1		33·7		33·8		27·2			7·7		13·6		12·2		13·8		14·7		
Current	28·7		54·8		47·2		50·2		60·1			5·3		21·9		13·4		18·9		30·2		
Missing	1·6		2·4		2·4		2·1		2·5			1·2		1·8		1·6		1·4		2·3		
Drinking status																						
Never	15·4		17·4		14·3		16·4		19·2		<0·001	57·7		43·7		45·5		44·1		42·2		<0·001
Former	5·7		4·2		4·2		3·7		4·4			2·4		3·5		2·9		3·8		3·8		
Current	78·2		77·7		81·0		78·8		75·7			39·0		51·9		50·9		51·1		53·1		
Missing	0·7		0·7		0·5		1·2		0·7			0·9		0·9		0·7		1·0		0·9		
Education																						
Junior high school	20·2		14·5		13·5		15·7		14·6		<0·001	19·7		12·7		11·0		13·6		13·6		<0·001
High school	49·3		52·0		51·0		51·0		52·8			49·6		52·4		50·0		53·6		53·6		
Junior college and vocational school	12·1		16·3		17·2		16·6		15·7			23·6		28·5		31·8		27·1		26·8		
College graduates and higher	16·6		15·5		16·8		15·0		15·1			5·3		4·9		5·8		4·4		4·4		
Others	1·8		1·8		1·5		1·6		1·9			1·7		1·5		1·4		1·3		1·6		
Annual household income (thousand Japanese yen)																						
0–2990	35·9		35·0		34·5		36·8		34·7		<0·001	40·2		42·9		39·4		45·7		44·1		<0·001
3000–5990	37·2		39·7		38·6		38·6		40·5			31·0		32·6		34·7		32·6		30·9		
6000–8990	15·2		15·3		17·0		14·1		14·8			11·5		11·7		12·9		10·1		11·5		
≥9000	7·9		6·3		6·7		6·6		6·0			6·6		6·2		6·9		5·9		5·9		
Missing	3·8		3·7		3·2		4·0		3·9			10·6		6·7		6·2		5·7		7·6		
Late bedtime	44·8		67·5		65·8		66·6		68·6		<0·001	60·9		79·8		80·4		80·5		79·1		<0·001
Missing	0·4		0·5		0·5		0·2		0·6			0·4		0·5		0·5		0·3		0·5		
Living alone	5·4		11·5		10·4		11·8		12·0		<0·001	6·4		9·5		9·1		9·3		9·9		<0·001
Health examinations in the last year	78·2		68·2		74·4		66·0		66·0		<0·001	79·4		70·4		75·5		70·7		66·3		<0·001
Missing	2·7		3·0		3·0		3·2		3·0			2·7		2·6		2·0		2·3		3·1		

Late bedtime included 11 p.m. or later and irregularly.

The *P*-values for the difference were tested between eating breakfast and three categories of skipping breakfast.

Table 2 shows sex-specific age- and area-adjusted and multivariable OR and 95 % CI of dietary behaviours compared with individuals who ate breakfast every day. In each analysis, only a small proportion of the participants with missing data were excluded (see online Supplemental Table 1). The age- and area-adjusted OR of eating out and eating instant foods frequently were significantly higher among individuals who skipped breakfast than those who ate breakfast. Even after adjusting further for education level, drinking status, smoking status, income and health examinations, these results remained statistically significant: the multivariable OR for skipping breakfast were 2·08 (95 % CI (1·96, 2·21)) in males and 2·15 (95 % CI (1·99, 2·33)) in females for eating out, and 1·89 (95 % CI (1·77, 2·01)) in males and 1·72 (95 % CI (1·56, 1·89)) in females for eating instant foods. The points estimates of the corresponding multivariable OR for overeating and eating quickly were lower than those for eating out and eating instant foods: 1·01 (95 % CI (0·96, 1·06)) in males and 1·10 (95 % CI (1·04, 1·15)) in females for overeating and 1·06 (95 % CI (1·01, 1·11)) in males and 1·07 (95 % CI (1·02, 1·13)) in females for eating quickly, respectively. These OR did not differ materially according to the frequency of skipping breakfast among responses of sometimes, often and usually.

**Table 2:** OR and 95 % CI of eating behaviours according to the frequency of breakfast intake

The frequency of skipping breakfast	Males										Females									
	Eating breakfast		Skipping breakfast		Sometimes		Often		Usually		Eating breakfast		Skipping breakfast		Sometimes		Often		Usually	
	(0/week)				1–2 /week		(3–4 /week		(≥5/week		0/week				1–2 /week		3–4 /week		(≥5 /week	
No. of participants^*^	42 999		8953		2402		1640		4911		53 179		7654		2576		1793		3285	
Overeating																				
	*n*	%	*n*	%	*n*	%	*n*	%	*n*	%	*n*	%	*n*	%	*n*	%	*n*	%	*n*	%
No. of yes	22 167	52	4823	54	1317	55	876	54	2630	54	32 676	62	4953	65	1688	66	1183	66	2082	64
	OR		OR	95 % CI	OR	95 % CI	OR	95 % CI	OR	95 % CI	OR	95 % CI	OR	95 % CI	OR	95 % CI	OR	95 % CI	OR	95 % CI
Age- and area-adjusted OR	1·00		0·87	0·83, 0·92	0·93	0·85, 1·01	0·88	0·80, 0·98	0·84	0·79, 0·90	1·00		1·03	0·98, 1·09	1·08	0·99, 1·17	1·10	0·99, 1·21	0·97	0·90, 1·05
Multivariable OR	1·00		1·01	0·96, 1·06	1·03	0·94, 1·12	1·01	0·91, 1·12	1·01	0·94, 1·07	1·00		1·10	1·04, 1·15	1·10	1·01, 1·20	1·15	1·04, 1·27	1·06	0·98, 1·15
Eating quickly																				
	*n*	%	*n*	%	*n*	%	*n*	%	*n*	%	*n*	%	*n*	%	*n*	%	*n*	%	*n*	%
No. of fast	21 800	51	5006	56	1336	56	904	55	2766	56	22 581	43	3590	47	1202	47	883	49	1505	46
	OR		OR	95 % CI	OR	95 % CI	OR	95 % CI	OR	95 % CI	OR	95 % CI	OR	95 % CI	OR	95 % CI	OR	95 % CI	OR	95 % CI
Age- and area-adjusted OR	1·00		0·99	0·95, 1·04	1·01	0·93, 1·10	0·99	0·89, 1·09	0·99	0·93, 1·05	1·00		1·06	1·01, 1·12	1·06	0·98, 1·15	1·18	1·07, 1·29	1·01	0·94, 1·08
Multivariable OR	1·00		1·06	1·01, 1·11	1·05	0·97, 1·15	1·05	0·95, 1·16	1·07	1·00, 1·14	1·00		1·07	1·02, 1·13	1·05	0·97, 1·14	1·19	1·08, 1·31	1·03	0·95, 1·10
Eating out																				
	*n*	%	*n*	%	*n*	%	*n*	%	*n*	%	*n*	%	*n*	%	*n*	%	*n*	%	*n*	%
No. of ≥3 times/week	5597	13	2456	28	655	27	486	30	1315	27	3187	6	1136	15	405	16	257	15	474	15
Age- and area-adjusted OR	1·00		2·05	1·93, 2·17	2·06	1·87, 2·27	2·35	2·10, 2·62	1·94	1·81, 2·09	1·00		2·25	2·09, 2·43	2·43	2·17, 2·72	2·19	1·91, 2·52	2·14	1·93, 2·38
Multivariable OR	1·00		2·08	1·96, 2·21	2·09	1·90, 2·31	2·41	2·15, 2·69	1·97	1·83, 2·12	1·00		2·15	1·99, 2·33	2·38	2·12, 2·66	2·11	1·83, 2·43	2·00	1·79, 2·23
Eating instant foods																				
	*n*	%	*n*	%	*n*	%	*n*	%	*n*	%	*n*	%	*n*	%	*n*	%	*n*	%	*n*	%
No. of ≥3 times/week	4020	9	1965	22	504	21	396	24	1065	22	1948	4	704	9	224	9	157	9	323	10
	OR		OR	95 % CI	OR	95 % CI	OR	95 % CI	OR	95 % CI	OR	95 % CI	OR	95 % CI	OR	95 % CI	OR	95 % CI	OR	95 % CI
Age- and area-adjusted OR	1·00		2·07	1·95, 2·21	1·99	1·79, 2·21	2·46	2·18, 2·77	2·00	1·85, 2·16	1·00		2·00	1·82, 2·19	1·92	1·66, 2·22	1·93	1·62, 2·29	2·10	1·85, 2·38
Multivariable OR	1·00		1·89	1·77, 2·01	1·89	1·69, 2·10	2·23	1·98, 2·52	1·78	1·64, 1·93	1·00		1·72	1·56, 1·89	1·80	1·55, 2·08	1·65	1·39, 1·97	1·70	1·49, 1·94

* Individuals with missing data of each behaviour were excluded from their analysis (see online Supplemental Table 1).

Multivariable OR were adjusted for age, area, education level, drinking status, smoking status, annual household income and health examinations.

The proportions of late bedtime and living alone were higher in individuals who skipped breakfast compared with those who ate breakfast (Table 3). The multivariable OR for skipping breakfast *v*. eating breakfast were 1·85 (95 % CI (1·75, 1·95)) in males and 1·98 (95 % CI (1·86, 2·11)) in females for late bedtime and 2·37 (95 % CI (2·17, 2·58)) in males and 2·02 (95 % CI (1·83, 2·21)) in females for living alone. However, the proportions of late bedtime and living alone did not differ according to the frequency of skipping breakfast among responses of sometimes, often and usually.

**Table 3:** OR and 95 % CI of breakfast-skipping-related lifestyles according to the frequency of breakfast intake

The frequency of skipping breakfast	Males										Females									
	Eating breakfast		Skipping breakfast		Sometimes		Often		Usually		Eating breakfast		Skipping breakfast		Sometimes		Often		Usually	
	(0/week)				(1–2 /week)		(3–4 /week)		(≥5 /week)		(0/week)				(1–2 /week)		(3–4 /week)		(≥5 /week)	
No. of participants^*^	42 999		8953		2402		1640		4911		53 179		7654		2576		1793		3285	
Late bedtime																				
	*n*	%	*n*	%	*n*	%	*n*	%	*n*	%	*n*	%	*n*	%	*n*%	%	*n*	%	*n*	%
No. of late bedtime	19 249	45	6042	68	1581	66	1092	67	3369	69	32 396	61	6111	80	2071	81	1443	81	2597	79
	OR		OR	95 % CI	OR	95 % CI	OR	95 % CI	OR	95 % CI	OR		OR	95 % CI	OR	95 % CI	OR	95 % CI	OR	95 % CI
Age- and area-adjusted OR	1·00		1·73	1·64, 1·82	1·67	1·53, 1·84	1·75	1·56, 1·95	1·75	1·64, 1·87	1·00		1·93	1·81, 2·05	2·05	1·85, 2·27	2·05	1·82, 2·32	1·78	1·63, 1·95
Multivariable OR	1·00		1·85	1·75, 1·95	1·75	1·60, 1·93	1·88	1·68, 2·11	1·88	1·76, 2·02	1·00		1·98	1·86, 2·11	2·03	1·83, 2·26	2·11	1·86, 2·39	1·87	1·71, 2·06
Living alone																				
	*n*	%	*n*	%	*n*	%	*n*	%	*n*	%	*n*	%	*n*	%	*n*	%	*n*	%	*n*	%
No. of living alone	2317	5	1031	12	250	10	194	12	587	12	3425	6	725	9	233	9	166	9	326	10
	OR		OR	95 % CI	OR	95 % CI	OR	95 % CI	OR	95 % CI	OR	95 % CI	OR	95 % CI	OR	95 % CI	OR	95 % CI	OR	95 % CI
Age- and area-adjusted OR	1·00		2·71	2·49, 2·94	2·39	2·08, 2·75	2·73	2·33, 3·20	2·87	2·59, 3·17	1·00		2·32	2·12, 2·53	2·13	1·84, 2·46	2·18	1·85, 2·58	2·56	2·25, 2·90
Multivariable OR	1·00		2·37	2·17, 2·58	2·20	1·90, 2·54	2·37	2·02, 2·79	2·46	2·21, 2·73	1·00		2·02	1·83, 2·21	2·02	1·74, 2·34	1·88	1·58, 2·24	2·10	1·84, 2·39

* Individuals with missing data of each behaviour were excluded from their analysis (see online Supplemental Table 1).

Late bedtime included 11 p.m. or later and irregularly.

Multivariable OR were adjusted for age, area, education level, drinking status, smoking status, annual household income and health examinations.

## Discussion

This large population-based cross-sectional study of Japanese males and females was, to our knowledge, the first to observe that individuals who skipped breakfast were more likely to have dietary behaviours of frequent eating out and frequent intake of instant foods and the lifestyle habits of late bedtime and living alone compared with those who did not, these findings were similarly observed for both males and females.

Our result showed that both males and females who skipped breakfast were more likely to be smokers and to undergo fewer health examinations than those who ate breakfast every day, which was consistent with a previous report that individuals who skip breakfast seemed to have poorer self-rated health and to pay less attention to health, as well as to have less knowledge of nutrition^(19)^. As for dietary behaviours related to breakfast intake, eating styles such as overeating and eating quickly may need to be considered, but our results indicated that these eating behaviours were only weakly related to breakfast skipping.

Previous studies, furthermore, reported that the main reasons for not eating breakfast were not having enough time^(20)^ and that the main reason for preferring fast foods or ready-to-eat foods was the convenience to eat^(21,22)^. A qualitative study also noted that the motivation for choosing food items in a busy schedule was convenience, quick and easy meal preparation^(23)^. Therefore, the consciousness of time and effort for eating as well as knowledge of health and nutrition could be important determinants of eating breakfast.

The higher probability of late bedtime among individuals who skipped breakfast in the present study is supported by previous studies that note the latest midpoint of sleep using bedtimes and rise times^(24)^ and shorter sleep duration (<6 h)^(25)^ with skipping breakfast. Individuals with late bedtime could have disturbed circadian rhythms leading to skipping breakfast because of poor or no appetite.

We also hypothesised that living alone may lead to skipping breakfast, based on systematic review showing that adults living alone were likely to have insufficient intakes of foods and nutrients compared with those living with others^(26)^. A previous study showed that dietary patterns in males aged 65 to 74 years who live alone had a higher proportion of poor dietary quality such as energy intake of less than two-thirds the RDA and less variety and nutrient intake, compared with those living with spouse^(27)^. This might be due to the lower energy intake because of skipping breakfast, because persons living alone aged 55 and older had a higher proportion of skipping breakfast compared with those living with a spouse^(28)^. In Japan, persons living alone aged ≥65 years, especially males, were more likely to skip meals (daily meal frequency ≤2/day)^(29)^. Our result was consistent with the findings of previous studies that individuals who lived alone seemed to have a poor dietary pattern, such as skipping breakfast.

The major strengths of our study were that it was a large population-based study for middle-aged and older adults, and thus, our finding could be generalised to other Japanese population. To the best of our knowledge, it is the first study to provide information that other dietary behaviours and lifestyles factors differ between those who eat breakfast and those who skip breakfast. Our study, however, has the limitation that the cross-sectional data used in the present study did not allow for the inference of causality. Second, since information on dietary behaviours was obtained using a self-administered questionnaire. The frequency of eating breakfast was likely affected by the subjective nature. It is possible that overeating and eating quickly could not be accurately assessed due to the subjective nature of the responses.

Our findings on different dietary and lifestyles factors between those who eat breakfast and those who skip breakfast could be of use in formulating nutrition policies involving comprehensive approaches for lifestyle modification that discourage eating late at night and encourage earlier bedtimes and waking early in the morning to improve the lifestyle patterns, rather than simply recommending the eating of breakfast.

## Conclusions

In conclusion, we found that individuals with a habit of skipping breakfast were more likely to eat out and to have the dietary habit of eating instant foods and lifestyle factors such as living alone and late bedtime than those who ate breakfast among Japanese middle-aged and older adults.

## Supplementary material

For supplementary material accompanying this paper visit https://doi.org/10.1017/S1368980023000010. click here to view supplementary material
