# Supplementary material for: Dietary behaviours and related lifestyles according to the presence or absence of skipping breakfast in Japanese adults: the JPHC-NEXT study
Source: Public Health Nutr. 2023 Feb 13;26(6):1230–7. doi: 10.1017/S1368980023000010 (PMC10346075; doi:10.1017/S1368980023000010)
Supplement: Supplementary file 1 [file S1368980023000010sup001.docx]

| **Supplemental Table 1.** Sex-specific distribution (%) of eating behaviors, and non-dietary outcomes according to the frequency of breakfast intake | | | | | | | | | | | |  |
| --- | --- | --- | --- | --- | --- | --- | --- | --- | --- | --- | --- | --- |
|  | Males | | | | |  | Females | | | | | |
|  | Eating breakfast | Skipping breakfast | | | *P*-value |  | Eating breakfast | Skipping breakfast | | | *P*-value | |
| (The frequency of skipping breakfast) |  | sometimes | often | usual |  |  |  | sometimes | often | usual |  |  |
|  | (0/wk) | (1-2/wk) | (3-4/wk) | (≥5/wk) |  |  | (0/wk) | (1-2/wk | (3-4/wk) | (≥5/wk) |  |  |
| **Overeating** |  |  |  |  |  |  |  |  |  |  |  | |
| Yes | 51.6 | 54.8 | 53.4 | 53.6 | 0.004 |  | 61.4 | 65.5 | 66.0 | 63.4 | <0.001 | |
| No | 47.7 | 44.3 | 45.8 | 45.6 |  |  | 37.7 | 33.9 | 33.5 | 35.8 |  | |
| Missing | 0.7 | 0.9 | 0.8 | 0.8 |  |  | 0.9 | 0.6 | 0.6 | 0.8 |  | |
| **Eating quickly** |  |  |  |  |  |  |  |  |  |  |  | |
| Very fast | 10.8 | 12.0 | 11.0 | 15.0 | <0.001 |  | 7.3 | 8.5 | 10.9 | 11.4 | <0.001 | |
| Slightly fast | 39.9 | 43.6 | 44.1 | 41.3 |  |  | 35.2 | 38.2 | 38.4 | 34.4 |  | |
| Normally | 40.2 | 35.0 | 37.2 | 36.2 |  |  | 46.7 | 42.2 | 40.0 | 41.0 |  | |
| Slightly slow | 7.8 | 8.0 | 6.8 | 6.3 |  |  | 9.7 | 10.2 | 9.4 | 11.2 |  | |
| Very slow | 1.1 | 1.0 | 0.7 | 1.0 |  |  | 0.9 | 0.7 | 1.1 | 1.8 |  | |
| Missing | 0.2 | 0.4 | 0.1 | 0.2 |  |  | 0.2 | 0.2 | 0.2 | 0.2 |  | |
| **Eating out** |  |  |  |  |  |  |  |  |  |  |  | |
| < 1 time/mo | 30.3 | 16.9 | 18.4 | 21.4 | <0.001 |  | 36.3 | 23.0 | 24.9 | 26.4 | <0.001 | |
| 1-3 times/mo | 39.7 | 33.7 | 30.8 | 31.9 |  |  | 42.3 | 38.3 | 38.4 | 40.2 |  | |
| 1-2 times/wk | 15.9 | 21.5 | 20.6 | 19.1 |  |  | 13.7 | 22.4 | 21.6 | 17.7 |  | |
| 3-4 times/wk | 5.2 | 11.4 | 17.0 | 9.9 |  |  | 3.5 | 9.1 | 9.1 | 7.3 |  | |
| 5-6 times/wk | 3.6 | 9.6 | 6.8 | 7.5 |  |  | 1.2 | 4.5 | 3.3 | 3.9 |  | |
| Every day | 4.2 | 6.2 | 5.9 | 9.4 |  |  | 1.4 | 2.1 | 2.0 | 3.3 |  | |
| Missing | 1.1 | 0.6 | 0.6 | 0.7 |  |  | 1.7 | 0.6 | 0.7 | 1.3 |  | |
| **Eating instant foods** |  |  |  |  |  |  |  |  |  |  |  | |
| < 1 time/mo | 24.0 | 10.9 | 10.1 | 12.1 | <0.001 |  | 37.2 | 21.7 | 23.6 | 25.9 | <0.001 | |
| 1-3 times/mo | 42.3 | 34.2 | 30.9 | 34.3 |  |  | 41.9 | 43.1 | 42.6 | 39.9 |  | |
| 1-2 times/wk | 23.5 | 33.5 | 34.3 | 31.2 |  |  | 15.6 | 25.8 | 24.6 | 23.2 |  | |
| 3-4 times/wk | 6.5 | 14.9 | 17.6 | 14.3 |  |  | 2.9 | 6.6 | 7.8 | 6.7 |  | |
| 5-6 times/wk | 1.6 | 4.4 | 4.6 | 4.4 |  |  | 0.5 | 1.7 | 0.8 | 2.0 |  | |
| Every day | 1.2 | 1.7 | 2.0 | 3 |  |  | 0.3 | 0.4 | 0.1 | 1.1 |  | |
| Missing | 0.8 | 0.3 | 0.6 | 0.8 |  |  | 1.5 | 0.7 | 0.5 | 1.2 |  | |
| **Bedtime** |  |  |  |  |  |  |  |  |  |  |  | |
| Before 7 p.m. | 1.2 | 0.7 | 0.8 | 0.9 | <0.001 |  | 0.4 | 0.6 | 0.2 | 0.5 | <0.001 | |
| 8 p.m. | 5.4 | 2.8 | 2.7 | 2.9 |  |  | 1.6 | 0.8 | 0.7 | 1.2 |  | |
| 9 p.m. | 19.5 | 10.0 | 10.1 | 9.1 |  |  | 10.1 | 3.6 | 4.9 | 4.9 |  | |
| 10 p.m. | 28.6 | 20.1 | 19.6 | 18.0 |  |  | 26.5 | 14.0 | 13.4 | 13.8 |  | |
| 11 p.m. | 27.9 | 28.6 | 27.9 | 26.5 |  |  | 38.7 | 35.9 | 33.7 | 30.2 |  | |
| 0 a.m. | 10.8 | 19.5 | 16.6 | 20.0 |  |  | 16.1 | 26.2 | 26.5 | 24.8 |  | |
| 1 a.m. | 2.5 | 6.2 | 6.9 | 9.2 |  |  | 3.5 | 9.5 | 9.7 | 10.7 |  | |
| 2 a.m. | 0.7 | 3.3 | 2.6 | 4.2 |  |  | 0.9 | 3.2 | 4.3 | 5.7 |  | |
| 3 a.m. | 0.2 | 0.6 | 1.2 | 1.4 |  |  | 0.1 | 0.4 | 0.8 | 2.1 |  | |
| 4 a.m. | 0.3 | 0.4 | 0.4 | 0.8 |  |  | 0.1 | 0.1 | 0.3 | 1.0 |  | |
| Irregularly | 2.4 | 7.2 | 11.0 | 6.4 |  |  | 1.3 | 5.0 | 5.1 | 4.5 |  | |
| Missing | 0.4 | 0.5 | 0.2 | 0.6 |  |  | 0.4 | 0.5 | 0.3 | 0.5 |  | |
| **Live in arrangement** |  |  |  |  |  |  |  |  |  |  |  | |
| Alone | 5.4 | 10.4 | 11.8 | 12.0 | <0.001 |  | 6.4 | 9.0 | 9.3 | 9.9 | <0.001 | |
|  |  |  |  |  |  |  |  |  |  |  |  | |
